# Exogenous spermidine alleviates oxidative damage and reduce yield loss in rice submerged at tillering stage

**DOI:** 10.3389/fpls.2015.00919

**Published:** 2015-10-31

**Authors:** Ming Liu, Meijie Chu, Yanfeng Ding, Shaohua Wang, Zhenghui Liu, She Tang, Chengqiang Ding, Ganghua Li

**Affiliations:** ^1^College of Agronomy, Nanjing Agricultural UniversityNanjing, China; ^2^Key Laboratory of Crop Physiology and Ecology in Southern China, Ministry of AgricultureNanjing, China; ^3^Jiangsu Collaborative Innovation Center for Modern Crop ProductionNanjing, China

**Keywords:** rice, submergence stress, exogenous spermidine, tillering stage, physiological characteristics

## Abstract

To figure out whether spermidine (Spd) can alleviate oxidative damage on rice (*Oryza sativa* L.) caused by submergence stress, Ningjing 3 was used in this study. The results showed that, spraying Spd on rice leaves at a concentration of 0.5 mM promoted the growth recovery of rice after drainage, such as green leaves, tillers, and aboveground dry mass. According to physiological analysis, Spd accelerate restored chlorophylls damage by submergence, and decreased the rate of $O_{2}^{\cdot -}$ generation and H_2_O_2_ content, inhibited submergence-induced lipid peroxidation. Spd also helped to maintain antioxidant enzyme activities after drainage, such as superoxide dismutase, peroxidase, and GR, which ultimately improved the recovery ability of submerged rice. With the effect of Spd, the rice yields increased by 12.1, 17.9, 13.5, and 18.0%, of which submerged for 1, 3, 5, 7 days, respectively. It is supposed that exogenous Spd really has an alleviate effect on submergence damage and reduce yield loss of rice.

## Introduction

Paddy flood disaster is one of China’s major agricultural disasters. The monsoon climate has a strong impact on the Yangtze River Basin and causes frequent flood disasters, which has brought serious threats to rice production safety. Therefore, the flood disaster has become one of the main obstacle factors limiting rice production in this area (Qin and Jiang, 2005; Huang and Qian, 2007). In the midstream and downstream of Yangtze River, panicles of rice is determined at tillering stage, when paddy fields often encounter flood disasters (Tian et al., 2005). As flood brings serious harm to the growth of rice in this period, effective measures should be took to reduce disaster losses.

Application of exogenous growth regulators is one of the effective ways to improve plant resistance of waterlogging. Studies have shown that paclobutrazol (Lin et al., 2006), proline and betaine (Hoque et al., 2007), brassinolide (BR; Li and Luo, 1999) and other exogenous regulators can effectively reduce the plant injury and yield loss caused by waterlogging. In recent years, polyamine (PAs) has been regarded as a new class of growth substances in improving plant stress resistance ability (Walters, 2003; Yuan et al., 2008). PAs are low-molecular-weight aliphatic amines that are ubiquitous in all organisms with high biological activity coming from biological metabolism. Common natural PAs include the higher PAs, spermine (Spm) and spermidine (Spd), and their diamine obligate precursor putrescine (Put). Among the three major PAs, Spd is most closely associated with stress tolerance in plants (Shen et al., 2000). Spd cannot only be used as a stress protective substance directly, but also be used as a signal molecule in stress signal transduction, constructing a stress resistant mechanism (Kasukabe et al., 2004). Spraying different concentrations of Spd on *Typha latifolia* L. could effectively improve the AsA and GSH content, GR and APX activity, and also reduce the production of active oxygen and MDA level in leaves under Cd^2+^ stress (Tang et al., 2005). Spd application to salinized nutrient solution resulted in an increase in PA and proline contents and antioxidant enzyme activities in cucumber seedlings, which contributed to osmotic adjustment during salinity (Duan et al., 2008).

It has been proved that exogenous Spd can improve plant resistance to drought (Németh et al., 2002), chilling (Zheng et al., 2008), aging (Wang et al., 2000), and heat (Tian et al., 2009), but whether exogenous Spd can be used to reduce submergence damage in rice has not been shown. To answer this question, whole plant of rice were harvested from both treated and untreated plants to measure growth, yield, and antioxidant enzyme activity. The present study aims to assess the possible effect of exogenous Spd application to alleviate the damage caused by submergence stress.

## Materials and Methods

### Experimental Design

The experiment was performed in 2014 growing seasons in Baolin village, Danyang County, Jiangsu province (31°54′N, 119°28′E). A japonica rice cultivar (Ningjing 3) popularized in lower reaches of the Yangtze River were grown on a farm during the rice growing seasons, which occurs from later-May to early-November. During the last year, the species was identified as sensitive to submergence. The soil was an Orthic Acrisol, with a total nitrogen content of 1.1 g kg^-1^, total phosphorus content of 0.48 g kg^-1^, total potassium content of 1.96 g kg^-1^. The experiment was arranged in a completely randomized block design with three replicates. The area of a plot was 4 m × 5 m = 20 m^2^.

### Crop Management

Seedlings 20-day-old raised in the seedling disk were transplanted in 10th June, with hill spacing of 0.3 m × 0.13 m and three seedlings per hill at both sites. Nitrogen (135 kg ha^-1^ N as urea), phosphorus (247 kg ha^-1^ P_2_O_5_ as single superphosphate), potassium (450 kg ha^-1^ K_2_O as KCl), were incorporated in plots 1 day before transplanting, additional N was applied 7 days after transplanting (135 kg ha^-1^), panicle initial (PI; 135 kg ha^-1^), and the stage of the second leaf from the top extension (135 kg ha^-1^).

The waterproof wall above 1 m was built around both sites, laying plastic film to prevent leakage. After 10 days transplanting, flooding treatment was started. The submerged water came from nearly river. Set 1, 3, 5, 7 days submergence treatments in four sites, respectively, and set a control site (CK) with no submergence. The submerged sites set a daily supplement of water to ensure complete submergence. At the end of the submergence treatments, excess water was discharged, and converted to normal field management.

Each submerged site was equally divided into two parts, separated by a ridge wrapped in plastic film. One part of each submerged site sprayed with Spd on rice leaves, which were signed as T1 + S, T3 + S, T5 + S, T7 + S, and the other part sprayed with equally water, signed as T1, T3, T5, T7, respectively. We used nebulizer to sprayed 50 ml Spd per square meter (0.5 mM, Sigma–Aldrich Chemical Co., St. Louis, MO, USA) on rice leaves at 8:00 and 18:00 in the day of drainage. Tween-20 (0.5%, v/v; Haijiechem, Zibo, China) was used both in Spd solution(T + S) and water(T) as a surfactant to increase adsorption, ensuring both side of the leaves were all stained with solution.

### Parameter Measurements

Four plants were sampled in each sites immediately at the day flooding over (0 day), and 3, 6, 9 days after spraying Spd. The fully expanded three leaves of the two plants immediately snap-frozen in liquid nitrogen, and stored at –40°C until required for analysis. The remaining two plants were oven-dried at 105°C for 30 min followed by 80°C for 72 h to constant weight. The tillers number and green leaves number per hill was counted by manual, as well as yield traits in mature stage.

Chlorophyll content was measured by the method according to a previous study (Li, 2000). The measurement of $O_{2}^{\cdot -}$ production rate has been described previously (Wang and Luo, 1990). H_2_O_2_ content was measured by the kit provided by Nanjing Jiancheng Biology Company. The leave sample (0.3 g) was homogenized with a mortar and pestle in 5 mL ice-cold phosphate buffer (50 mM, pH 7.8) containing 1% (w/v) insoluble polyvinylpolypyrrolidone (PVPP; Jiang and Zhang, 2002). The extract was centrifuged at 16,000 × *g*_n_ for 20 min at 4°C. The supernatant was used to measure enzyme activity. Superoxide dismutase (SOD) activity, peroxidase (POD) activity and GR activity was measured according to the method of Li (2000). Malondialdehyde (MDA) content was determined by the thiobarbituric acid reaction following the method described previously (Zhao et al., 1994).

### Data Analysis

All data were analyzed by SPSS (IBM SPSS statistics 20), and the results were presented as the means ± SD. Statistically analyzed using Duncan’s multiple range test at a level of significance of 0.05. Figures were manufactured by Microsoft Excel 2007 software.

## Results

### Effects of Exogenous Spd on Growth and Yield of Submerged Rice

#### Growth

**FIGURE 1:**
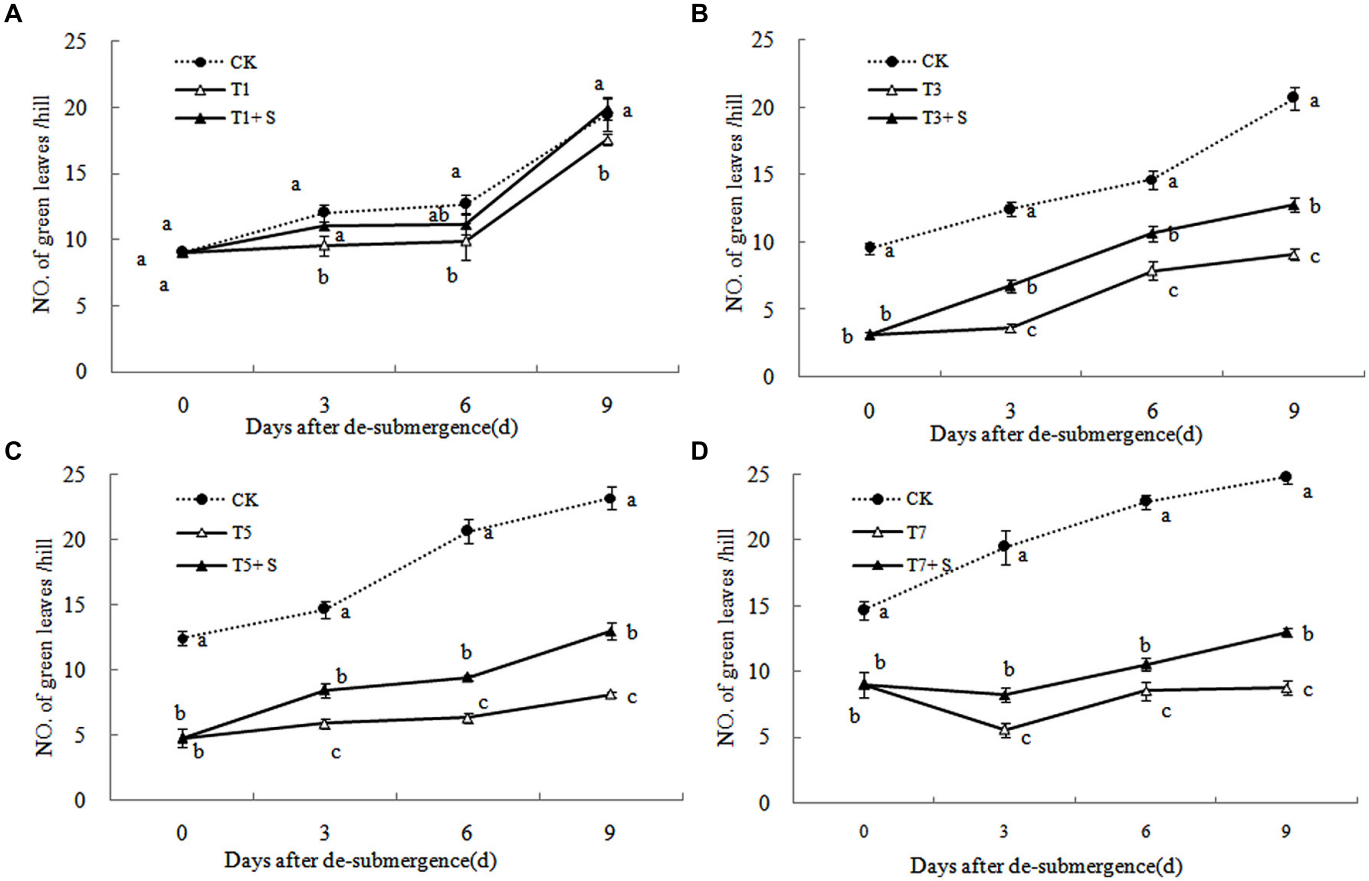
**Effects of spermidine (Spd) on green leaves number per hill of rice after submergence.** CK indicate control with no submergence, T1, T3, T5, T7 indicate submerged for 1, 3, 5, 7 days, respectively. T1 + S, T3 + S, T5 + S, T7 + S indicate submerged for 1, 3, 5, 7 days and spray spermidine after drainage, respectively. The same as follow.

**FIGURE 2:**
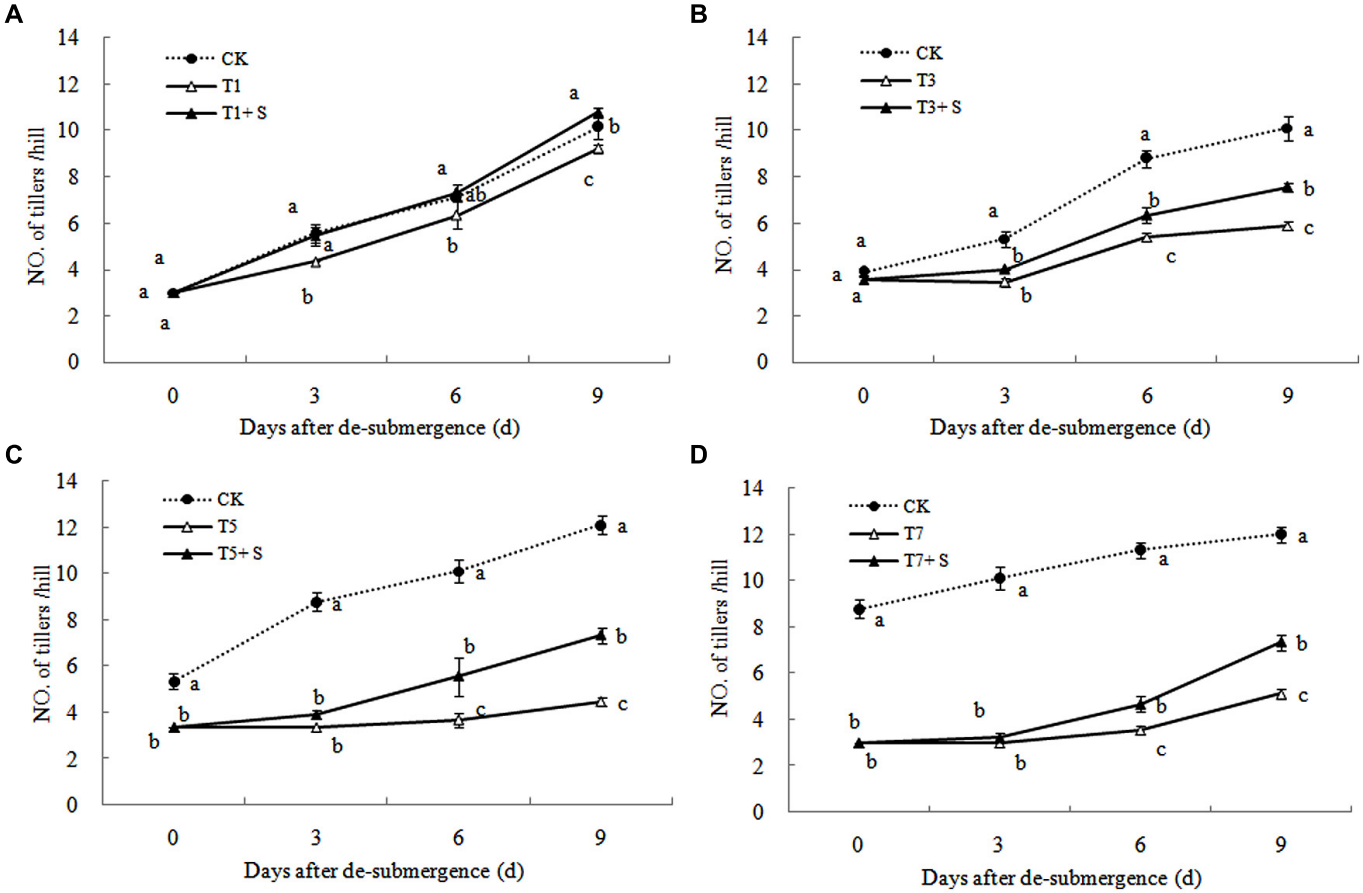
**Effects of Spd on tillers number per hill of rice after submergence**.

**FIGURE 3:**
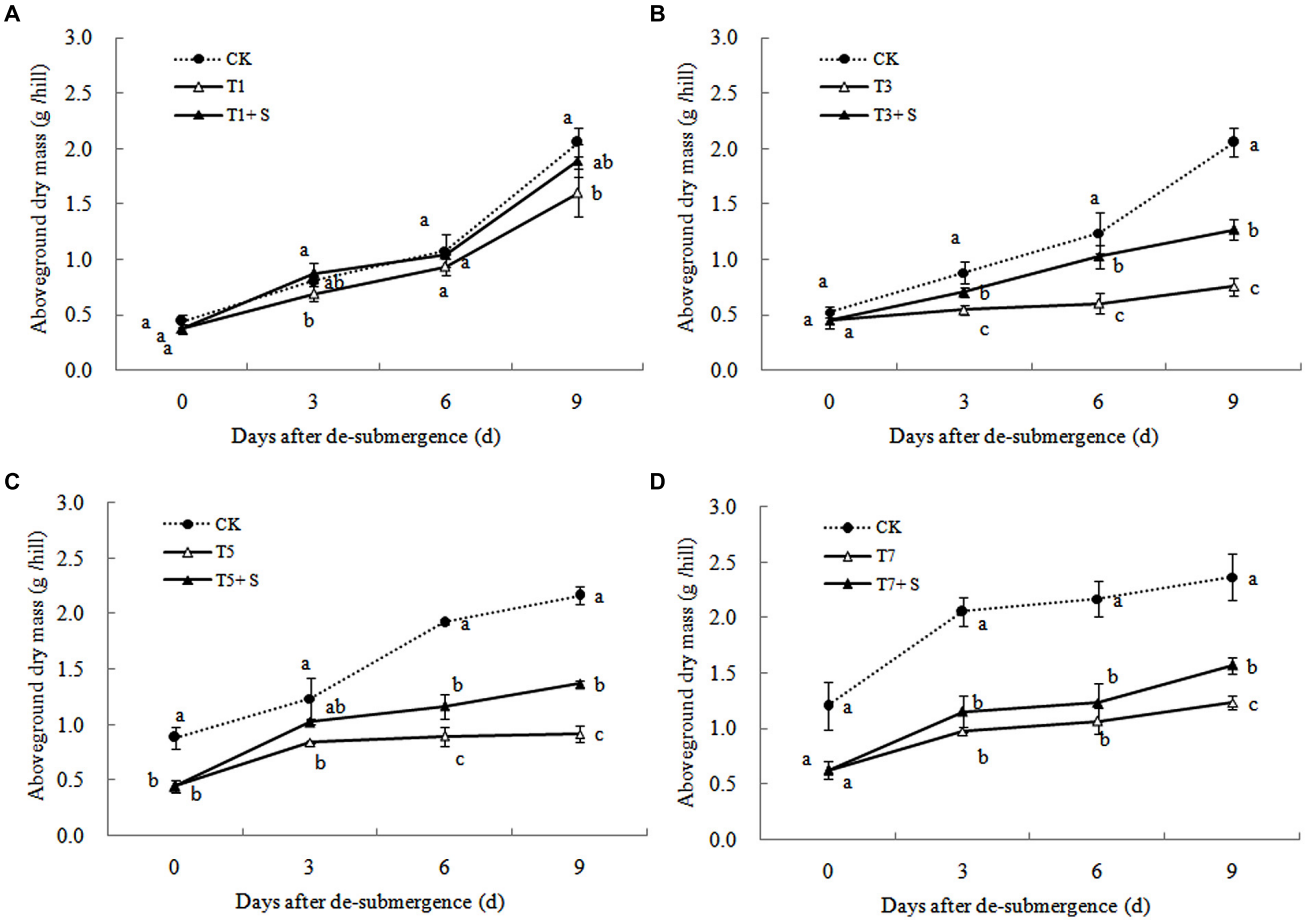
**Effects of Spd on shoot dry weight per hill of rice after submergence**.

Except for T1 treatment, the green leaves number (**Figure 1**), tillers number (**Figure 2**) and aboveground dry mass (**Figure 3**) of rice were significantly decreased after submergence stress (*P* < 0.05) compared with the control, and resumed slowly after drainage. Application of exogenous SPD significantly increased the green leaves number, tillers number, and aboveground dry mass of submerged rice, effectively alleviating the submergence-mediated growth reduction.

#### Yield

**FIGURE 4:**
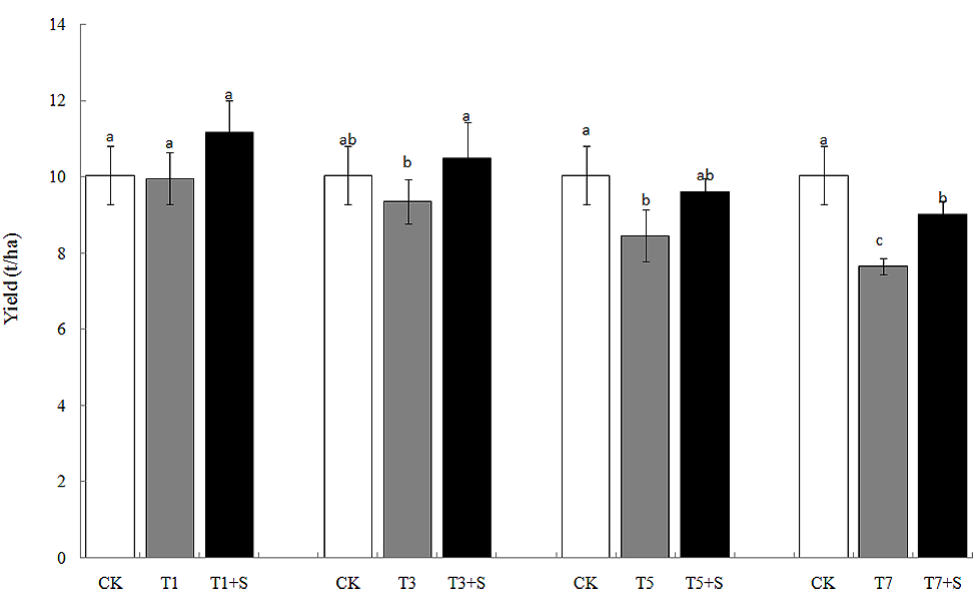
**Effects of Spd on yield of rice after submergence**.

The rice yields decreased gradually with the increase of the days under submergence stress (**Figure 4**), especially the yields of T5 and T7 significantly decreased compared with the control (CK). Exogenous Spd apparently elevated rice yields after submergence stresses.12.1 and 17.9% of yields were increased, respectively, compared with T1 and T3, and returned to the CK level. Exogenous Spd also increased the yields of T5 + S and T7 + S by 13.5 and 18.0%, respectively.

**FIGURE 5:**
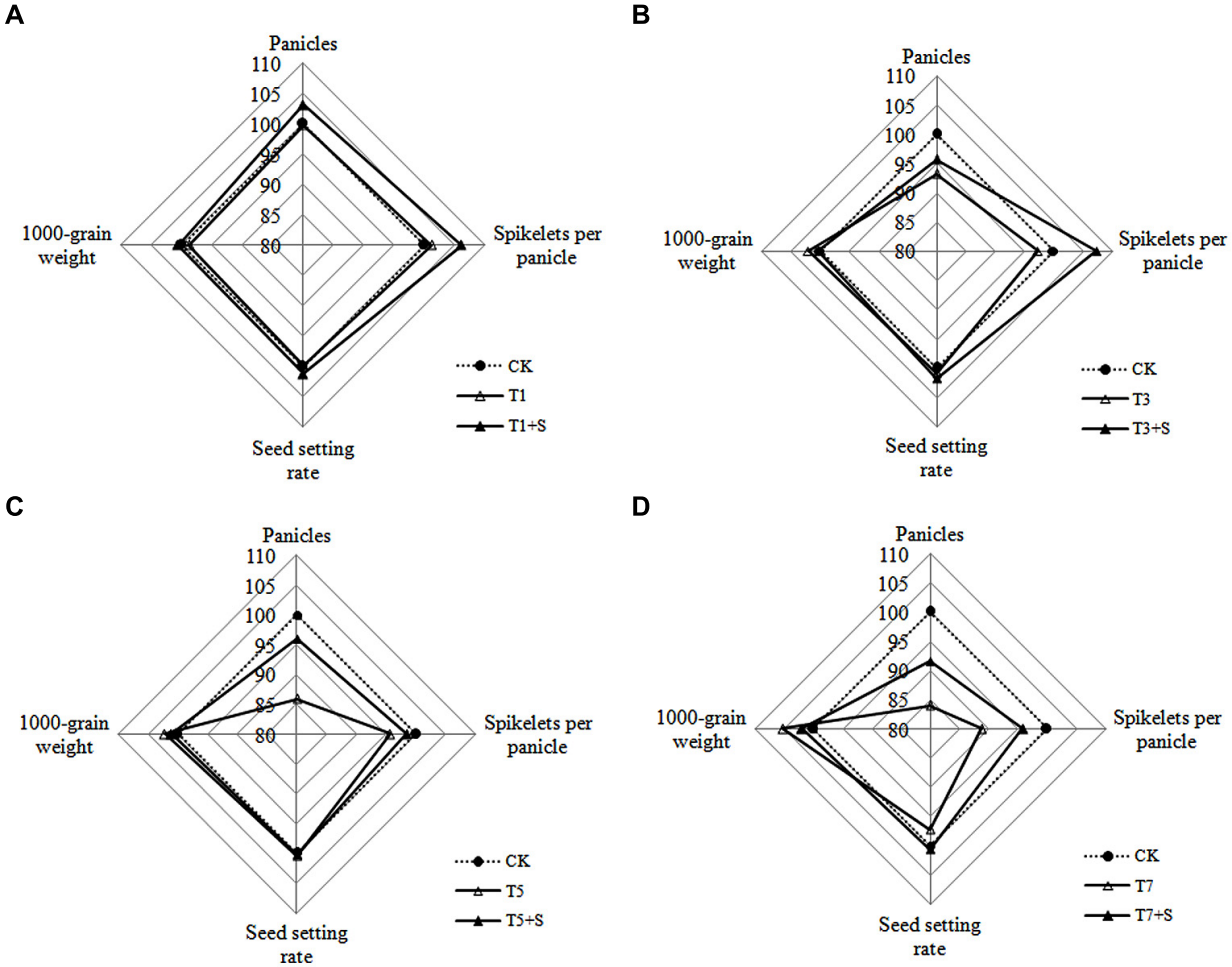
**Effects of Spd on yield traits of rice after submergence**. Each data point in the spider plots represents the percentage of the mean values of the agronomic traits. The mean measurements from the CK were assigned a 100% reference value.

As for yield traits (**Figure 5**), panicle number and spikelet number per panicle decreased due to submergence stresses, while the seed setting rates and 1000-grain weights were less affected. Application of exogenous Spd obviously improved the panicle number and spikelet number per panicle, not the seed setting rates and 1000-grain weights.

### Effects of Exogenous Spd on Physiological Characteristics of Submerged Rice

#### Chlorophyll Content

**FIGURE 6:**
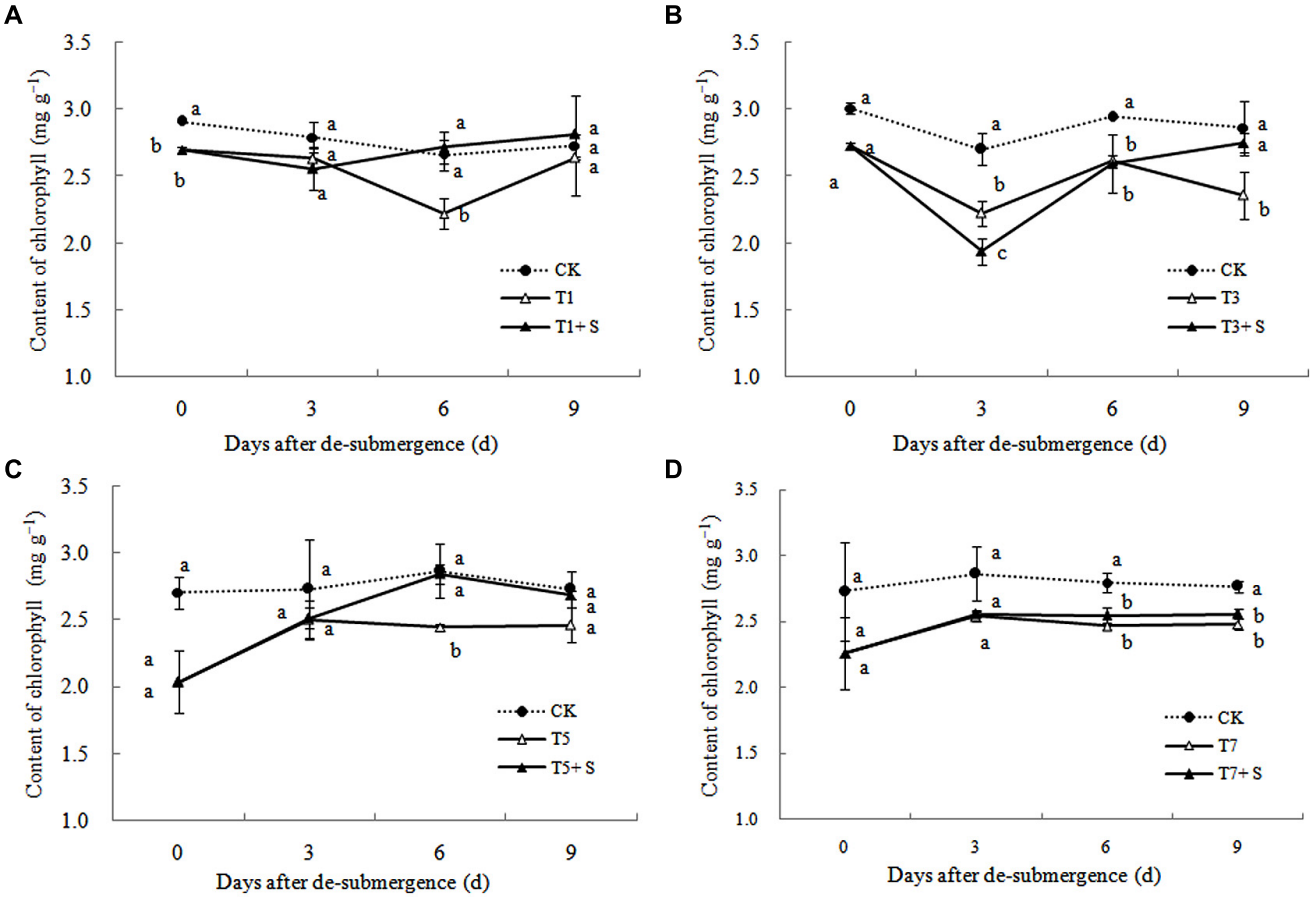
**Effects of Spd on chlorophyll content of rice after submergence**.

After different days of submergence treatment, the chlorophyll content of leaves under submergence conditions was significantly lower than that of leaves under CK conditions (**Figure 6**). The plants applied with exogenous Spd, had an accelerate effect on the process of chlorophyll recovery, but not apparently for T7 + S.

#### Free Radical Production and Membrane Damage

**FIGURE 7:**
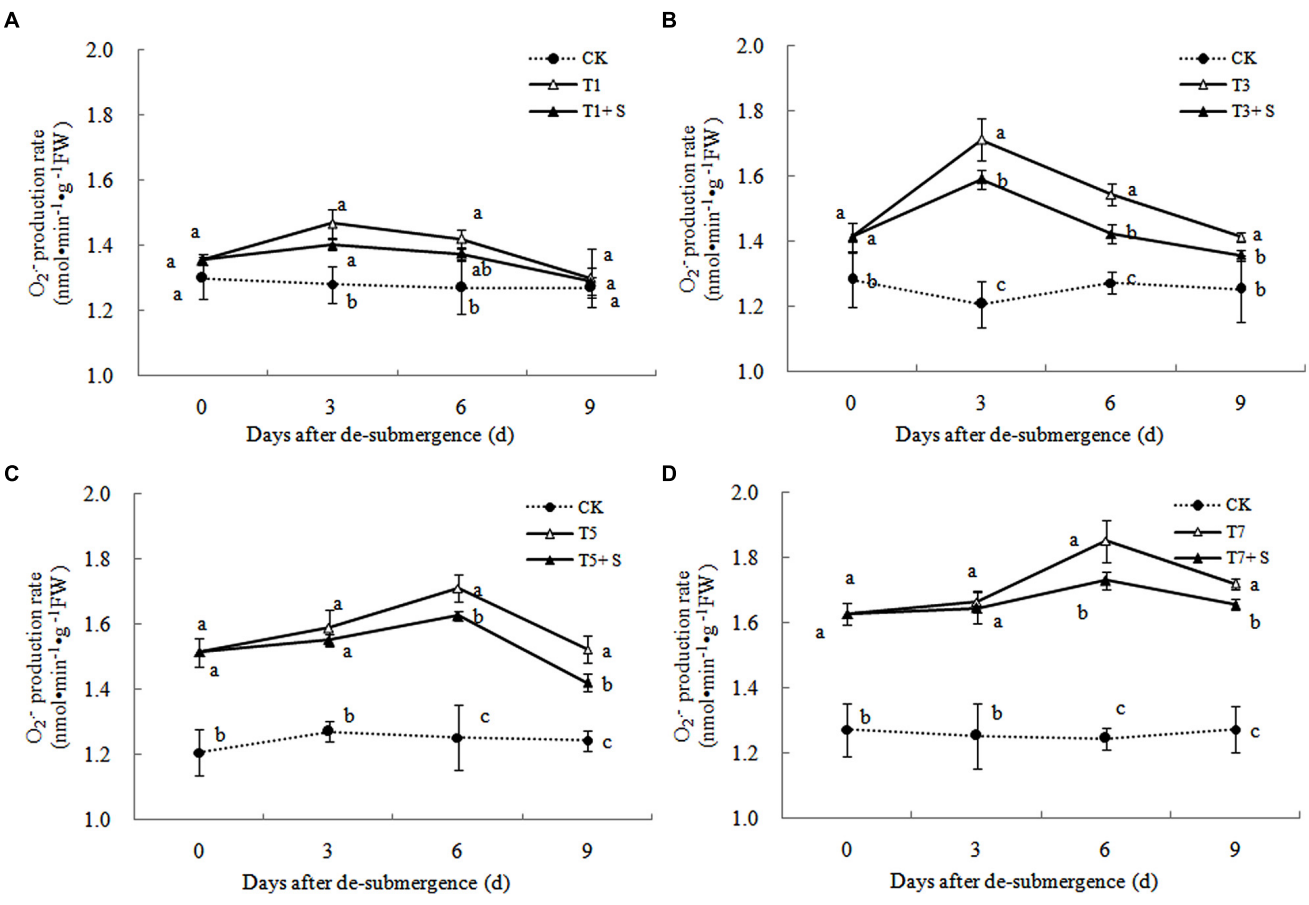
**Effects of Spd on $O_{2}^{\cdot -}$ production rate of rice after submergence**.

**FIGURE 8:**
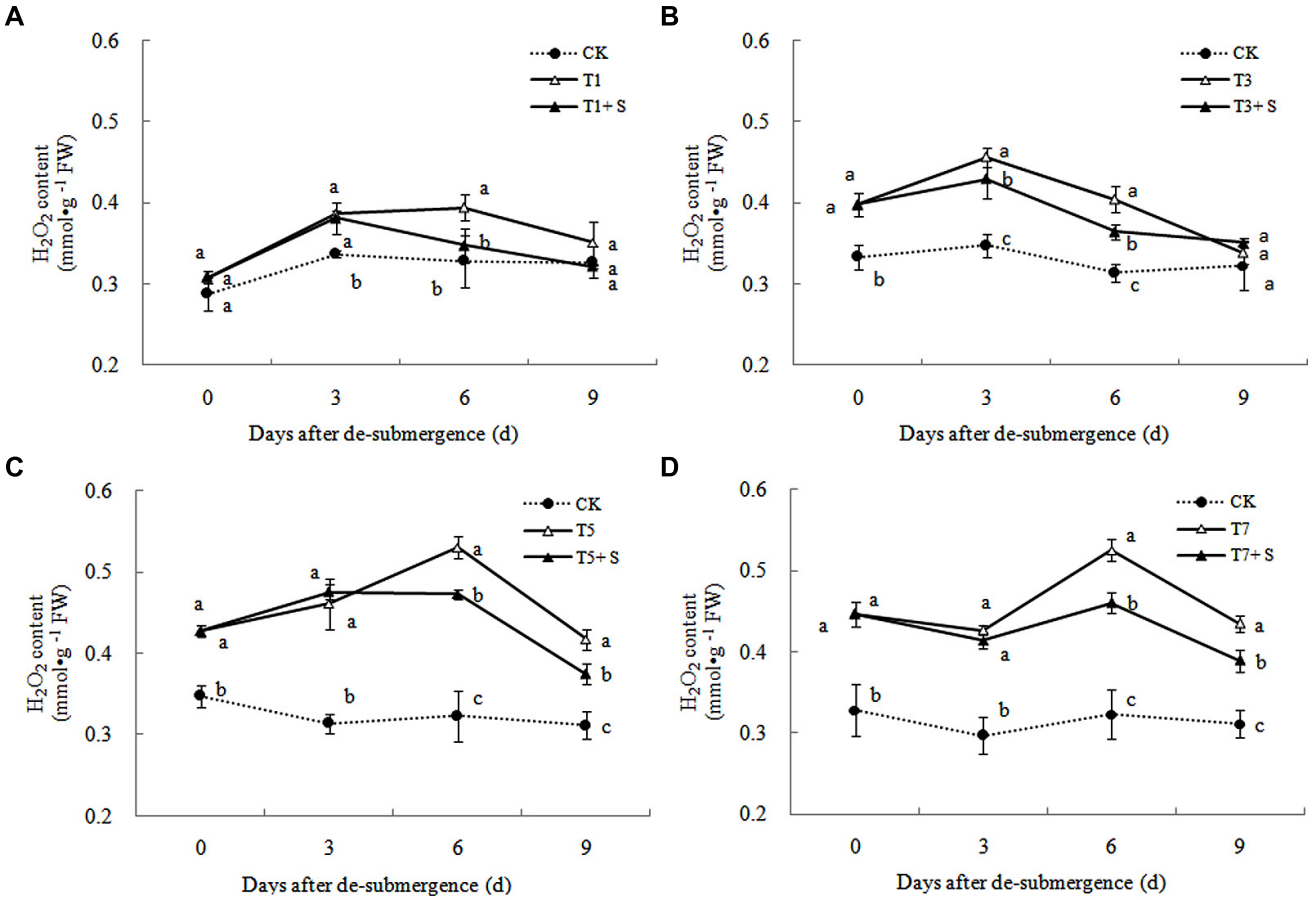
**Effects of Spd on H_2_O_2_ content of rice after submergence**.

**FIGURE 9:**
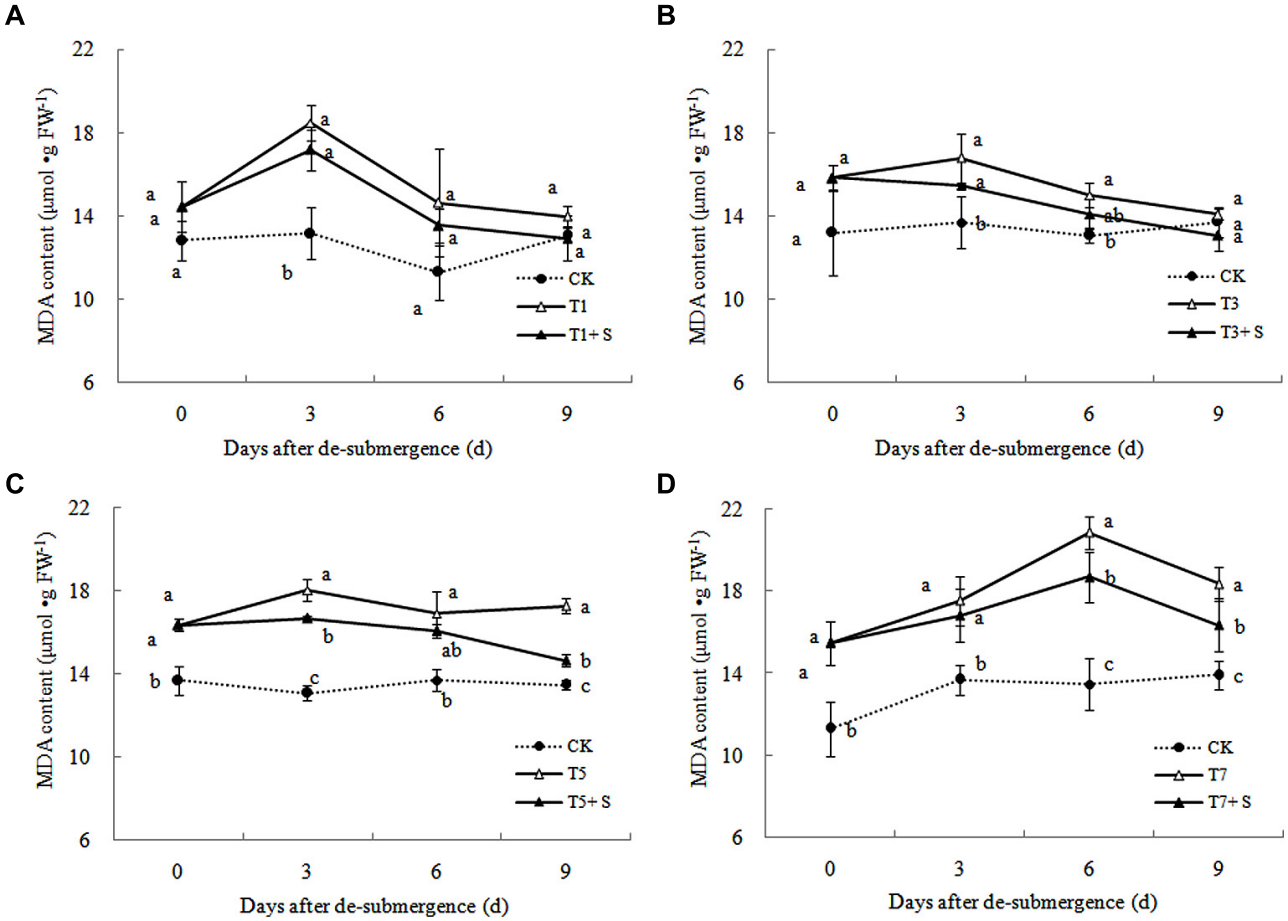
**Effects of Spd on malondialdehyde (MDA) content of rice after submergence**.

In plants subjected to submergence stress for 3 days or longer, the $O_{2}^{\cdot -}$ production rate (**Figure 7**) and H_2_O_2_ content (**Figure 8**) significantly increased compared with CK plants. After 9 days of recovery, the $O_{2}^{\cdot -}$ production rate and H_2_O_2_ content of T1 and T3 reduced to the CK level, exogenous Spd accelerated this process. This phenomenon could also found in T5 + S and T7 + S, the $O_{2}^{\cdot -}$ production rate were 6.7 and 3.8% lower than T5 and T7 at ninth day after drainage, and H_2_O_2_ content was 10.2 and 10.4% lower, respectively. Lipid peroxidation of membranes can be estimated from the MDA content. In submerged plants, MDA levels were significantly higher than those in the CK plants (**Figure 9**) at third day after drainage. The MDA contents, respectively, decreased by 7.5, 7.1, 15.2, and 11.0% than water sprayed plants (T), after 9 days of Spd treatments.

#### Antioxidant Enzyme Activity

**FIGURE 10:**
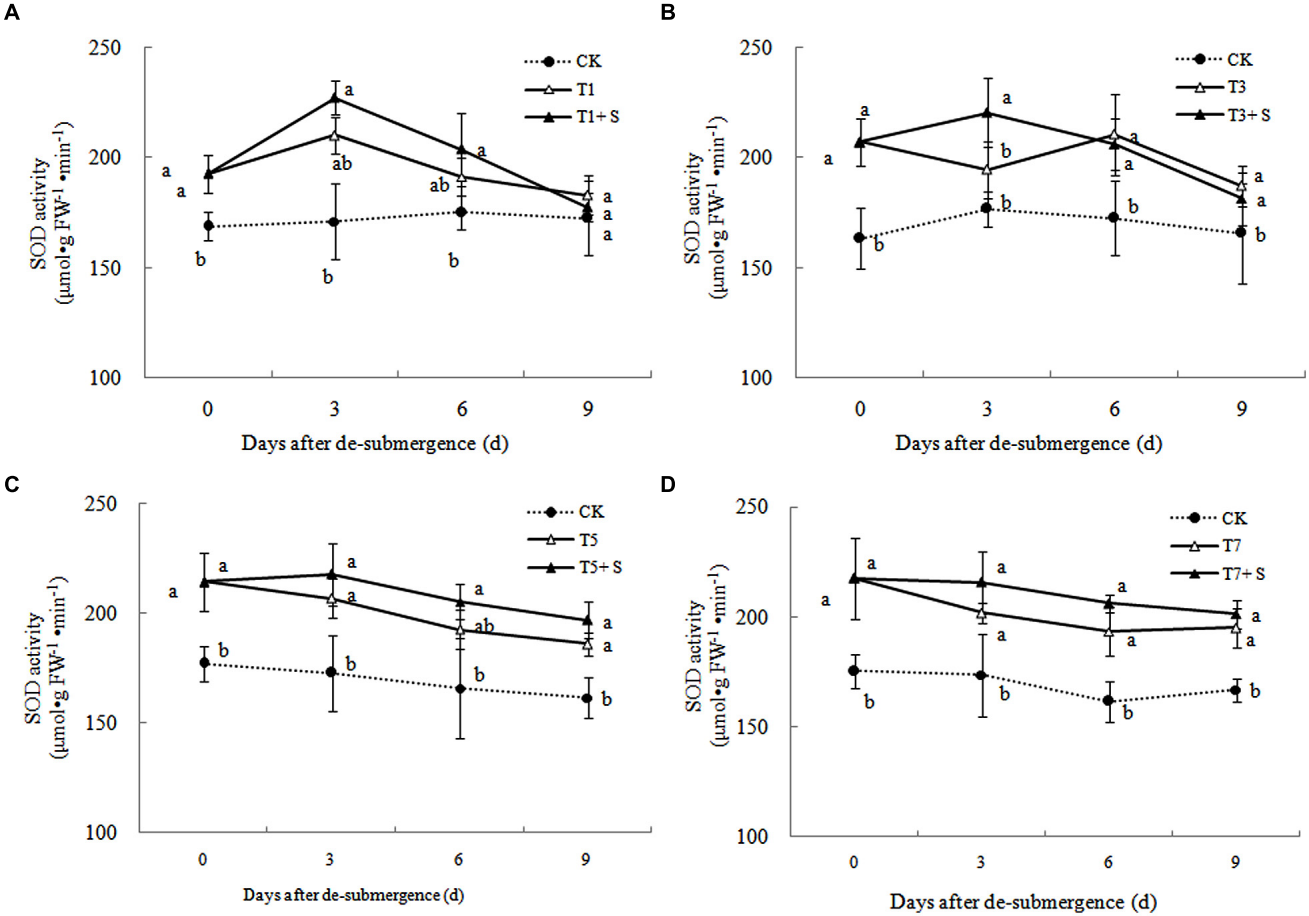
**Effects of Spd on superoxide dismutase (SOD) activity of rice after submergence**.

**FIGURE 11:**
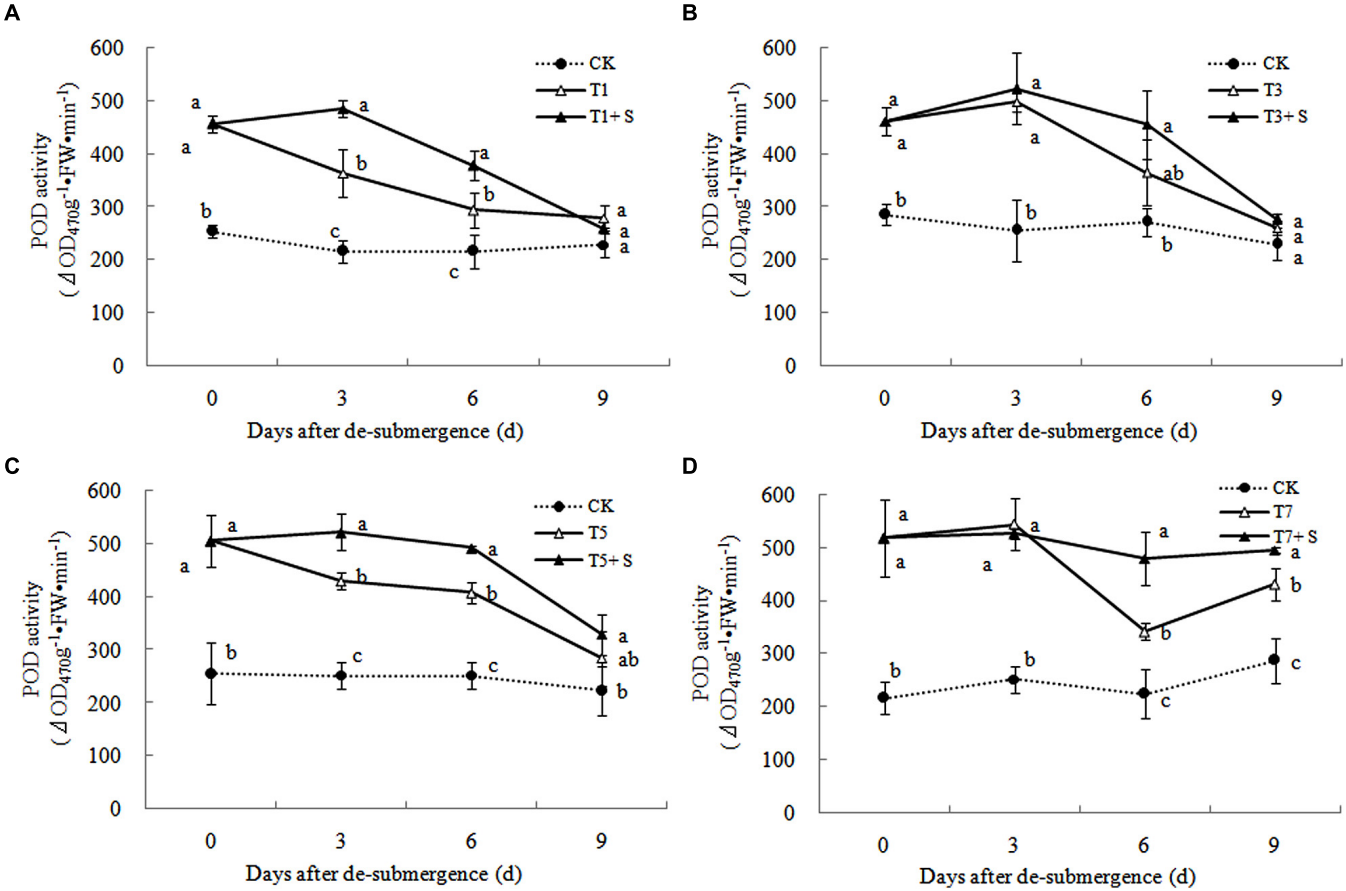
**Effects of Spd on peroxidase (POD) activity of rice after submergence**.

**FIGURE 12:**
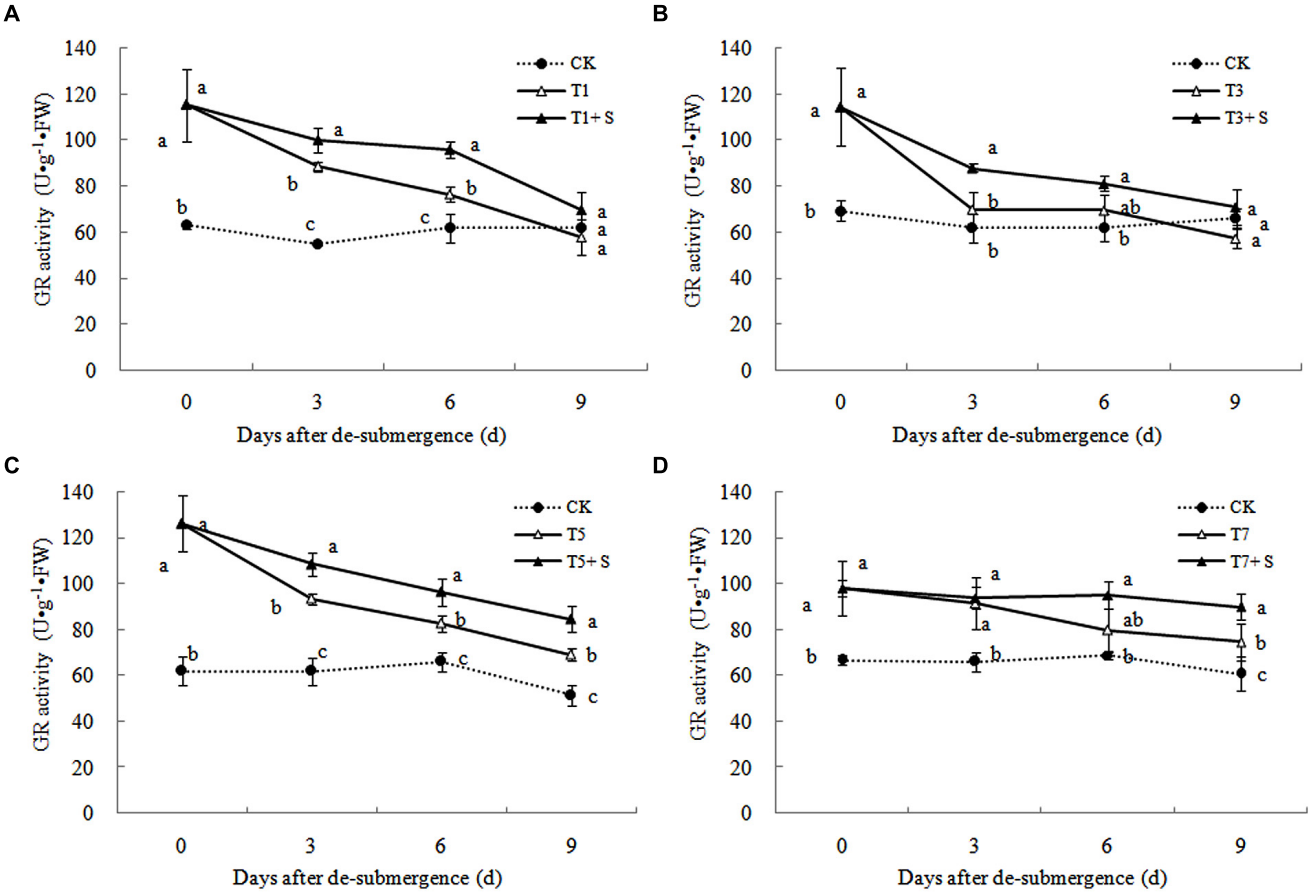
**Effects of Spd on GR activity of rice after submergence**.

Superoxide dismutase, POD, and GR were the enzymes selected to evaluate the oxidative damage caused by flooding on the antioxidant defense system. SOD activity was significantly enhanced after submergence stresses (**Figure 10**), and similar phenomenon were observed in POD (**Figure 11**) and GR (**Figure 12**). Whereas the activities of these enzymes turned to decline rapidly after drainage. We observed that exogenous Spd further increased the activity of SOD, 8.16, 13.31, 5.29, and 6.98% higher than T1, T3, T5 and T7 in third day after spraying, respectively. Similarly, POD and GR activities were also apparently elevated compared with the plants sprayed with water. In addition, exogenous Spd slowed down the decline of the activities of antioxidant enzymes which maintain high levels consistently.

## Discussion

Under natural conditions, plants maintain a balance between producing and scavenging ROS through a well-coordinated and rapidly responsive antioxidant system (Bowler et al., 1992). However, diverse environmental stresses differentially affect cellular homeostasis and induce the formation of ROS, such as submergence, which causes oxidative damage to membrane and lipids (Srivalli et al., 2003). MDA content can reflect the damage degree of plants, because lipid peroxidation of membranes can be estimated from the MDA content (Liao et al., 2005). In this study, submergence stress significantly increased the levels of $O_{2}^{\cdot -}$ (**Figure 7**) and H_2_O_2_ (**Figure 8**)_,_ as well as the MDA contents (**Figure 9**). These indicators increased at first and then decreased after drainage, showing that submergence stress caused a delayed stress on rice, which is similar to the research on the rapeseed (Tao, 2013). Rely on the recovery abilities themselves, the MDA content, $O_{2}^{\cdot -}$ production rate and H_2_O_2_ content in the plants of T1 and T3 can return back to the normal levels after drainage. However, it was not showed in T5 and T7, probably the activities of antioxidant enzymes was stronger affected (Han et al., 2011). It is well documented that PAs can counteract oxidative damage in plants by acting as direct free radical scavengers (Drolet et al., 1986). On the other hand, Spd may act as a protectant for the plasma membrane against stress damage by maintaining membrane integrity (Tadolini et al., 1984; Tadolini, 1988). Our results showed that exogenous Spd promoted the reduction of the severe lipid peroxidation under submergence stress (**Figures 7**–**9**), which is in agreement with the previous report (Yiu et al., 2009).

It has been reported that a major role of oxygen radicals in chlorophyll destruction by waterlogging in mung bean leaves is indicated (Ahmed et al., 2002). It was also found in this study that chlorophyll significantly decreased in rice leaves under submergence stress (**Figure 6**). Lower chlorophyll would turn the leaves yellow, resulting in a decline in photosynthesis and photosynthetic products, thus affects plant physiological metabolism (Zahed et al., 2009). Our results showed that chlorophyll losses were effectively reversed by the exogenous Spd (**Figure 6**), which was consistent with other study (Wang et al., 2000). The increased chlorophyll content in leaves perhaps has an important role to promote the growth of tillers and leaves and increase dry matter of rice (**Figures 1**–**3**).

The major ROS-scavenging enzymes of plants include SOD, POD, CAT, APX, and GR. SOD converts $O_{2}^{\cdot -}$ to H_2_O_2_ (Alscher et al., 2002), whereas POD, CAT, and GR help to minimize the damaging effects of H_2_O_2_ by converting it into oxygen and water (Peters et al., 1989; Liu et al., 2009). With the action of the enzymes, the MDA content, $O_{2}^{\cdot -}$ production rate and H_2_O_2_ content showed a trend of decline (**Figures 7**–**9**). Previous research indicated that exogenous Spd could increase antioxidant enzymes activities in plant under stress (Xu et al., 2001; Kasukabe et al., 2004; Tang and Newton, 2005). Our study found that exogenous Spd not only promoted the activities of SOD, POD, and GR, but also delayed the rate of decline in these enzymes activities (**Figures 10**–**12**). Maintaining a high level of activities can ensure the efficient removal of ROS (Huang et al., 1990; Jiang et al., 1992; Xie et al., 1998). The probably reason for Spd acting as an inhibitor of ROS production is that Spd can scavenge ROS directly (Drolet et al., 1986) or indirectly by improving antioxidant enzyme activities, through combining with antioxidant enzymes molecule (Slocum et al., 1984; Mehta et al., 1991). In this study, how Spd scavenge ROS is unclear, which may need further research to verify it.

Submergence stress causes adverse effects on plant growth and productivity (Boru et al., 2001; Wang et al., 2007). Growth analysis is widely used as a tool to characterize plant growth. Our results showed that exogenous Spd sprayed on rice leaves significantly alleviated the growth inhibition by submergence stress, increasing green leaves (**Figure 1**), tillers (**Figure 2**), and biomass accumulation (**Figure 3**).

Damage of submergence stress on rice was ultimately reflected on yield. Our results showed that the panicle number and the spikelet number per panicle of rice significantly decreased under submergence stress (**Figure 5**), leading to a decline of yield (**Figure 4**). The longer under submergence, the more yield decreased (Lin et al., 1997). Submergence stress seriously affected the growth of tillers (**Figure 2**), thus led to the significant decrease of the panicle number. The spikelet number per panicle are closely associated with nutrient levels at tillering stage (Mae, 1997), which is the reason why the spikelet number per panicle declined resulted from the decrease of biomass accumulation. The exogenous Spd significantly improved the panicle number, the spikelet number per panicle and grain yields of rice (**Figures 4** and **5**), probably resulting from the raising of the green leaves, tillers, and biomass accumulation (**Figures 1**–**3**). We found that spraying Spd increased rice yields by more than 10%, indicating that the exogenous Spd plays an important role in reducing the yield loss.

## Author Contributions

GL conceived and designed the experiments. YD and SW performed the experiments. ML analyzed the data. ML and MC wrote the paper.

## Conflict of Interest Statement

The authors declare that the research was conducted in the absence of any commercial or financial relationships that could be construed as a potential conflict of interest.
